# Pathogenic invasive microbes *Trichoderma pleuroticola* transform bacterial and fungal community diversity in *Auricularia cornea* crop production system

**DOI:** 10.3389/fmicb.2023.1263982

**Published:** 2023-11-03

**Authors:** Lei Ye, Bo Zhang, Lingzi Zhang, Xuezhen Yang, Wei Tan, Xiaoping Zhang, Xiaolin Li

**Affiliations:** ^1^Sichuan Institute of Edible Fungi, Chengdu, China; ^2^Department of Microbiology, College of Resources, Sichuan Agricultural University, Chengdu, China

**Keywords:** *Auricularia cornea*, *Trichoderma pleuroticola*, pathogenic invasive microbes, metagenome, fungi, bacteria, microbial diversity

## Abstract

Pathogenic invasion of *Trichoderma pleuroticola* profoundly altered microflora in the *Auricularia cornea* crop production system, impacting diversity and composition in both artificial bed-log and fruiting bodies. A more complex ecological network between the diseased and healthy bodies. Researchers still have poor knowledge about how the important agricultural relationship between the composition of the microbiome of the artificial bed-log and the fruiting bodies is infected by the pathogenic invasive microbes *T. pleuroticola*, but this knowledge is crucial if we want to use or improve it. Here, we investigated 8 groups (48 biological samples) across 5 growth stages of the *A. cornea* production system using metagenomic technology. Diseased and healthy fruiting bodies exhibited distinct microbial compositions, while core members in artificial bed-logs remained stable. Core microbiota analysis highlighted *Pseudomonas* and *Pandoraea* bacterial genera, as well as *Sarocladium*, *Cephalotrichum*, *Aspergillus*, and *Mortierella* fungal genera as biomarker species after the bodies were treated with the pathogenic invasive microbes *T. pleuroticola*. In diseased bodies, these core members upregulated pathways including polymyxin resistance, L-arginine degradation II, superpathway of L-arginine and L-ornithine degradation, glucose degradation (oxidative), glucose and glucose-1-phosphate degradation, promoting fruit spoilage. Our data confirm that *T. pleuroticola* plays an important role in the early stages of disease development in the *A. cornea* crop generation system. The exposed volatile core microbiome may play an important role in accelerating *T. pleuroticola*-induced decay of fruiting bodies.

## 1. Introduction

*Auricularia cornea* (Dai and Yang, 2018), a Basidiomycetes of the genus *Auricularia* classification into the Auriculariaceae, previously known as *A. polytricha* (Mont.) Sacc. until 2018. Globally recognized as “wood ear” or “jelly ear,” this white rot fungus gained swift popularity since its introduction to China in the 1980s. It has evolved into a prominent cultivated variety in Asia, notably favored for the ‘hot pot’ industry, contributing to China’s distinction as the world’s foremost *A. cornea* producer, with an annual yield of approximately 2.2 million tons. The production epicenter comprises Sichuan, Shandong, Fujian, and Henan provinces, collectively accounting for an impressive 89.85% of the total output. *A. cornea* thrives optimally within elevated temperature ranges, particularly between 25°C and 30°C. Accompanied by ample external water supply, it exhibits rapid growth under these conditions. However, this unique high-temperature, high-humidity environment inadvertently fosters the propagation of diseases and pests, thereby posing a notable challenge to quality cultivation. In previous studies, there are many reports on the occurrence of natural diseases of *A. cornea*, the most common of which is the “running ear” caused by a large amount of water supply in the growth period of the body, and the malformed ear of the fruiting body caused by insufficient oxygen. During the last 6 years, the commercial production of wood ear mushroom has encountered substantial impediments owing to outbreaks of brown rot epidemics, as previously detailed in our report (Ye et al., 2023). Besides, several studies have shown the most common diseases in the production of *A. cornea*, such as the “cob-web disease” caused by *Cladobotryum cubitense* (Wang et al., 2018, 2019) and *Hypomyces mycophilus* (Cao et al., 2023), “slippery scar” caused by *Scytalidium auricola* (Peng et al., 2013, 2014; Ye et al., 2023) and *Scytalidium lignicola* (Sun and Bian, 2012) species, and “brown rot disease” caused by *Trichoderma pleuroticola* (Ye et al., 2023) species. Overall, the occurrence of these diseases predominantly manifests through distinct lesions on artificial bed-log (a mixture consisting of *A. cornea* mycelium and substrate and contained in a plastic bag) and fruiting bodies. Unfortunately, the composition and structure of the fruited bodies and MCM microbial communities caused by *T. pleuroticola* have not been reported, but this knowledge is essential for the effective control of brown rot in production practice. Therefore, to further strengthen the study of microbial communities in artificial bed-log and fruiting bodies is conducive to better exhibit the panoramic of the microbial community caused by disease, improve the understanding of the disease invasion process, and provides data support for disease prevention and control. Just like the work we are doing right now.

“Brown rot,” a physiological disease, which seriously endangers the sustainable production of *A. cornea*. The pathogen *T. pleuroticola* was confirmed in previous studies, which can infect more than a dozen artificial fungi, among which *Ganoderma lingzhi*, *A. heimuer*, *A. cornea* and other crops, and seriously affected in China. Fungal pathogens are highly adapted microorganisms with the ability to cause disease, and they are adapted to the interaction of a particular microbe with a particular host. Since pathogens must overcome similar host barriers, common themes of microbial pathogenesis have developed. However, these mechanisms differ between species and are not necessarily conserved. *T. pleuroticola* is first described by Park et al. (2006), due to the seriously affected by green mold epidemics on oyster mushroom culture in Korea, and later found in soil and wood in Europe, North America, Peru, Iran and other countries (Komoń et al., 2007; Blaszczyk et al., 2013). The substrate infections with *T. pleuroticola* led to notable yield reductions in Poznań oyster mushrooms cultivation (Sobieralski et al., 2012). China reported its first occurrence of *T. pleuroticola* in 2013, isolated from the rotten cap of Asafoetida mushroom (Zhang et al., 2013). In our study, the pathogen sprayed was culturable, isolated from decaying *A. cornea* fungus, and confirmed through Koch’s postulates. Several studies have reported edible mushroom diseases caused by pathogenic invasive microorganisms *T. pleuroticola* in China. Three *T. pleuroticola* strains (TTP) were isolated from contaminated artificial bed-log of Zha Shui black fungus (*Auricularia auricula*) in Zhashui County, Shaanxi Province, China, and their sequence homology comparison showed that the TTP was clustered under the same clade with *T. pleuroticola* (accession number: JQ040377.1, strain number: CQJB5015), the homology reached 100%, and combined with the morphological characteristics of TTP were identified again. The main metabolites produced by *T. pleuroticola*, such as esters, ketones volatile organic compounds, amino acids and their derivatives, have antifungal activities, which may inhibit the mycelia growth of *Auricularia auricula* (Kong, 2022). A strain ZJ-03 isolated from oyster mushroom culture medium, identified by combining morphological observation and molecular biology as the *T. pleuroticola*, and producting xylanase activity above 3,000 IU/g (Qian et al., 2020), which considered an important source of xylanase production (Korkmaz et al., 2017). A study of enzyme activity of laccase from the extracellular secretion of *Trichoderma pleuroticola* showed that when reaction temperature was 25°C, pH value of succinic acid buffer was 4. 0，concentration of succinic acid buffer was 0. 045 M, and dosage of guaiacol was 0. 04 M, laccase activity of extracellular secretion of *Trichoderma* crude enzyme was the best (Zhang et al., 2021). *T. pleuroticola* can cause green mold epidemics of *Ganoderma lingzhi* and *Cyclocybe aegerita*, which can be biocontrolled by *Bacillus pumillus* (strain number JSW-0601) (Xie et al., 2018; An et al., 2022). Six genus *Trichoderma* species were isolated and identified based on ITS and morphological characteristics, from China’s shiitake planting region, including *T. pleuroticola*, *T. harzianum*, *T. atroviride*, *T. viride*, *T. longibrachiatum*, and *T. oblongisporum*. It was also proved that adjusting medium pH was not effective in preventing and controlling green mold disease (Wang et al., 2016). Overall, the *T. Pleuroticola* caused a variety of cultured mushroom diseases, and seriously threatened the artificial production. In addition, studies have shown that the genus *Trichoderma* fungi can produce a variety of compounds, such as peptide chains, terpenes, steroids, amides and cyclopentenone, as well as a variety of enzymes, such as lassase, cellulase and xylanase, which have biological activities, such as anti-tumor, anti-fungal, antibacterial, antiviral and bioinduced DPPH free radical scavenging, and are used for the production of enzymes and other metabolites. It is widely used in agriculture, papermaking, chemical industry and so on. Recently, the brown rot disease pandemic in *Auricularia cornea* crop production system in China, caused a serious threat for sustainable agriculture. However, the extent to which these pathogens can alter the microbiome of artificial bed-log and fruiting bodies remains poorly understood, complicating the prediction of future outbreaks. Such disease occurrences have a profound impact on cropping efficiency, yield, and quality.

Understanding the pathogenesis of invasive fungi and the response of the microbial community in crop production systems can help optimize the management of infections, and a better understanding of these processes can help managers develop better preventive measures. To analyze the effects of spraying spore suspensions of the pathogen on the bacterial and fungal communities in the fruiting bodies and culture medium, metagenomic sequencing methods were used here to study the characteristics of the microflora in the *A. cornea* culture production system. The primary objective of our research was to discern the disparities in microbial community composition between healthy and diseased samples in both the medium and fruiting bodies. This investigation aims to provide crucial data for the development of comprehensive strategies for disease prevention and control on a larger scale.

## 2. Materials and methods

### 2.1. Experimental treatments and sampling strategies

Samples from a subset of treatments called the greenhouse experiment, previously published by Ye et al. (2023) were used for this study. The experiment was conducted in Shifang, Sichuan Province, China (104°2′57′′E, 31°12′ 55′′ N) in April to July 2022. The test strains of *A. cornea* were identified based on the sequence ITS and artificially cultured in greenhouses. The culture medium was calculated as air-dried material and consisted of 10% cottonseed hull, 42% corncob, 42% sawdust, 2% cornmeal, 1% gypsum, and 3% lime. The moisture content of the medium was 65%. The medium was placed in a transparent polyethylene plastic bag with a length of 48 cm and a folding diameter of 20 cm, weighing 4.8 kg per bag. The medium was then autoclaved at 121°C for 2.5 h and inoculated with *A. cornea* spawn after cooling. After inoculation, mycelial culture was performed in a dark culture room at an ambient temperature of 25°C and relative humidity of 70%. When the fungal mycelium has penetrated the entire culture medium (FMBM), it is transferred to the greenhouse for bodies breed. Ambient temperature is 22°C to 28°C, humidity is 90 to 95%, natural light at midday on a clear day is 500 lux, and photoperiod is the same as natural conditions. The pathogenic invasive microbes *T. pleuroticola* were confirmed in a previous study (Ye et al., 2023). Here, PDA medium (glucose 20 g, peptone 5 g, agar 20 g, magnesium sulfate 0.5 g, potassium dihydrogen phosphate 1 g, water 1,000 mL) was used to culture *T. pleuroticola* on cellophane, spores were picked up with a sterile inoculation trench and added to sterile water to make a suspension, and the spore volume was 10^5^/mL for pathogenic spraying of fruiting bodies.

To determine whether *T. pleuroticola* was an endogenous or exogenous pathogen of *A. cornea*, samples of the medium and mycelial mixture (MCM) and fruiting bodies at different growth stages were collected throughout the experiment. The experimental design was a randomized complete block experiment with 8 periods and three replicates. A total of 48 samples were collected, 24 of which were used for bacterial community detection and 24 for fungal community detection. Briefly, the first MCM sample was collected in the FMBM period, and subsequent samples were collected every 7 days and named as FUL, PRI, OPE, and MAT, respectively. Observation of the population samples showed that the fruiting bodies of *A. cornea* at the MAT stage had no disease characteristics and were healthy. Then, the artificial bed-log were divided into 6 groups (30 bags per group placed in a 1 m^2^ planting area), of which 3 groups were treated with spore suspension (TS) and the remaining 3 groups were treated with sterile water (CK). To clarify the influence of *T. pleuroticola* on the microflora in the fruiting bodies and MCM of *A. cornea*. Immediately afterwards, 200 mL/m^2^ of the spore suspension of the pathogen was sprayed in the TS group and the same amount of sterile water was sprayed in the CK group after sampling at the MAT stage. 7 days later, the fruiting body and MCM samples of the groups TS and CK were collected. After briefly, rinsing the fruiting bodies with sterile water, the lesion area was sampled with a 5 mm diameter perforator in the TS group and also in the CK group and numbered FBE and ZCE, respectively. MCM samples were collected on a clean bench according to aseptic procedures and numbered with FBL and ZCL. Approximately 10 g of MCM or fruiting bodies were collected and placed in a sterile 15 mL tube, immediately placed in liquid nitrogen for 30 s, and then stored at −80°C until used for microbial diversity analysis.

### 2.2. DNA extraction and sequencing

DNA was extracted from the 48 biological samples using the FastDNA SPIN kit (MP Biomedicals, OH, United States) following the manufacturer’s instructions. The quality of DNA extraction was verified by 0.8% agarose gel electrophoresis, and DNA was quantified using a UV spectrophotometer. The extracted DNA was amplified by PCR with the specific primers (F: ACTCCTACGGAGGCAGCA and R: GGACTACHVGGGTWTCTAAT) targeting the hypervariable regions V3 to V4 of the 16S rRNA gene. For fungal amplification, the region ITS was selected for analysis with primers F: GGAAGTAAAAGTCGTAACAAGG and R: GCTGCGTTCTTCATCGATGC. For PCR amplification, Q5 high-fidelity DNA polymerase from NEB was used, and the number of amplification cycles was strictly controlled (Zhang et al., 2022). PCR amplification products were detected by 2% agar-gel electrophoresis, and target fragments were cut and recovered using the gel recovery kit of AXYGEN. After the preliminary quantitative results of electrophoresis, the recovery products of PCR amplification were quantified by fluorescence. The Quant-iT PicoGreen dsDNA Assay Kit was used as the fluorescent reagent and the Microplate Reader (BioTek, FLx800) as the quantitative instrument. The TruSeq Nano DNA LT Library Prep kit from Illumina was used to prepare the sequencing libraries. Prior to computer sequencing, the library was run on the Agilent Bioanalyzer using the Agilent High Sensitivity DNA kit. Then, the Quant-iT PicoGreen dsDNA Assay Kit was used to quantify the library on the Promega QuantiFluor Fluorescence Quantification System, and the qualified library concentration was above 2 nM. Illumina MiSeq (Illumina, United States) sequencer was used for 2 × 300 bp paired-end sequencing with MiSeq Reagent kit V3 (600 cycles) (Zhang et al., 2022). The optimal sequencing length of the target fragments was 200 bp to 450 bp. The raw sequencing data were saved as FASTQ (FQ) flies. The Illumina MiSeq bacterial sequence raw sequences were entered into the National Center for Biotechnology Information (NCBI) Sequence Read Archive database with the ID bioproject as PRJNA994454. The Illumina MiSeq sequence data from fungi were entered into the NCBI with the ID bioproject as PRJNA994467.

### 2.3. Bioinformatic analyses

Microbiome bioinformatics was performed using QIIME 22019.4 with slight modifications according to the official tutorials^1^ (Bolyen et al., 2019). Briefly, raw sequence data were demultiplexed using the demux plugin and primers were cut using the cutadapt plugin. Sequences were then quality filtered, denoised, merged, and chimeras removed using the DADA2 plugin (Callahan et al., 2016). The feature table after singularity elimination was statistically analyzed, and the distribution of the composition of each sample in the six classification levels phyla, class, order, family, genus, and species was visualized, and the analysis results were plotted in a bar graph. A tree of microbial taxonomic rank was drawn in the form of a tree map, and the abundance of each amplicon sequence variants (ASV) group was added to the diagram in the form of a pie chart (Carrión et al., 2019). The Chao 1 (Chao, 1984), Shannon (Shannon, 1997), Simpson, Observed species, Pielou’s evenness, and Good’s coverage Alpha diversity metrics were used to comprehensively assess the diversity of microbial communities, a boxplot was drawn using R based on the ASV date, and Dunn’s test was used as a *post hoc* test to show the differences in alpha diversity between groups. A boxplot was created using R to show the differences in alpha diversity between the different groups, and was tested using Dunn’s test. The Shannon rarefaction curve was used to show the sequencing depth. At the ASV level, the distance matrix of each sample was calculated, and the difference and significance of beta diversity between different groups were measured by a series of unsupervised sorting and clustering methods combined with appropriate statistical testing procedures. The Silva database (Release 132, http://www.arb-silva.de) for the 16S rRNA gene of bacteria was used for annotation of species. ITS sequences of fungi were annotated using the UNITE database (Release 8.0, https://unite.ut.ee/). The QIIME2 classify – sklearn algorithm (Bokulich et al., 2018) was used.^2^ For each feature sequence of ASVs, species annotations are generated using a pre-trained Naive Bayes classifier using default parameters in QIIME2 software. QIIME2 was used to smooth the ASVs feature sequences for further analysis. The MetagenomeSeq method was used for differential analysis of metabolic pathways, and the fitFeatureModel function was called to fit the distribution of each metabolic pathway with zero-inated log-normal model, and the fitting results of this model were used to detect the significance of differences and to find out the metabolic pathways with significant differences between groups. At the species taxonomic composition level, a variety of unsupervised and supervised ranking, clustering, and modeling methods were used in combination with appropriate statistical testing procedures to measure differences in species abundance composition among different groups, and to find marker species. Based on the distribution of species composition in each sample, the correlation network was constructed (SparCC algorithm), the topological index was calculated, and the keystone species were identified. The Zi and Pi score values of each node in the network were calculated using R, and the role of each node in the associated network was determined based on the score (Deng et al., 2012). Based on the results of 16S rRNA and ITS gene sequencing, microbial metabolic function was predicted using PICRUSt2 (Phylogenetic Investigation of Communities by Reconstruction of Unobserved States) using MetaCyc, KEGG, and COG database annotations, various metabolic pathways were identified, and species composition of specific metabolic pathways was determined. All statistical analyzes were performed using IBM SPSS statistics version 22.0.0. ANOVA data were presented as mean ± standard deviation (±SD) of each sample group, *n* = 3, *p* < 0.05 in LSD test.

## 3. Results

### 3.1. Epidemic characteristics of pathogen contamination by *T. Pleuroticola*

**Figure 1:**
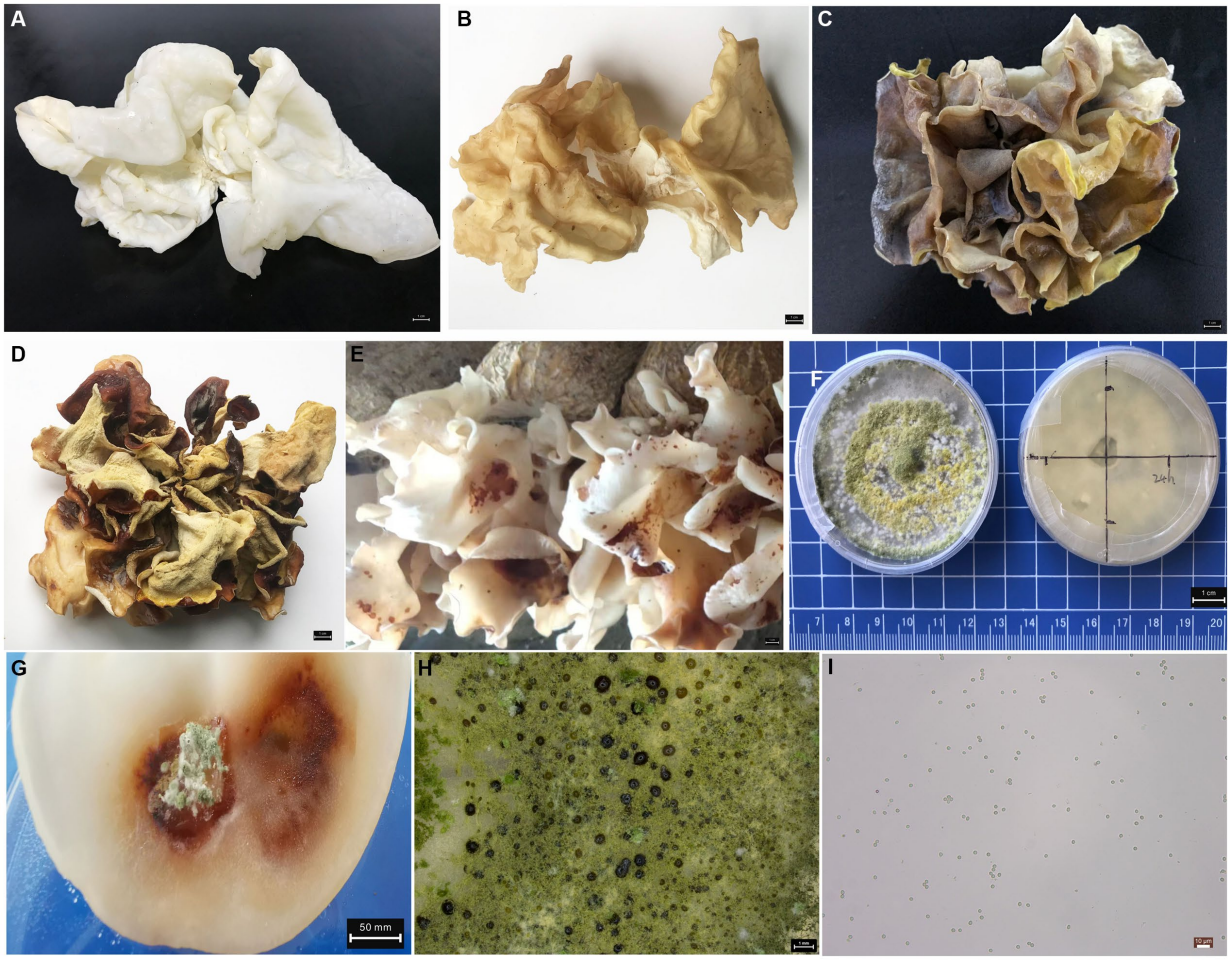
Morphological characteristics of fruiting bodies and pathogenic fungi **(A)** healthy fresh fruiting bodies. **(B)** Healthy air-dried fruiting bodies. **(C)** Disease-fresh fruiting bodies. **(D)** Disease-dried fruiting bodies. **(E)** Early characteristics of fruiting body disease under greenhouse cultivation. **(F)** Pathogens of *T. pleuroticola* were grown on PDA medium. **(G)** disease fruiting bodies in Koch’s postulate test. **(H)** Morphology of *T. pleuroticola* colony, scale 1 mm. **(I)** Spore morphology of *T. pleuroticola*, scale 10 μm.

Pure white *A. cornea* strain is widely cultivated in Asia for its good appearance, flavor and widely culinary value (Figures 1A,B). In a previous note, we first reported the characteristics of *A. cornea* crop disease caused by *T. pleuroticola* (Ye et al., 2023). The 596 bp ITS sequence of *T. pleuroticola* was deposited in GenBank under accession number ON974844.1. As we described previously, the bodies disease caused by *T. pleuroticola* show a distinct yellow sediment with a darker shape (Figures 1C,D), and up to 20% of the natural occurrence in greenhouse cultivation. Due to the occurrence of the disease, the fruit body loses its commercial value in a short time. After 84 h of spraying with the pathogenic fungal spore suspension, the fruiting bodies showed distinct disease characteristics (Figure 1E), with blotchy lesions and brown appearance. The pathogenic invasive microbes *T. pleuroticola* were confirmed by testing Koch’s postulates (Figures 1F–I). The growth rate of *T. pleuroticola* was 13.92 ± 1.24 mm/day on PDA medium at 25°C. This produces a large number of spores in a short time, which spread in the environment. The rapid spread of spores could be the main reason for further aggravation of field diseases. Under natural environmental conditions, infected fruiting bodies exhibited significant decay and foul odor after 120 h, which could be due to interactions with other microorganisms in the environment. We further performed metagenomic sequencing to elucidate the characteristics of the microbial communities of the fruit bodies and artificial bed-log during the *T. pleuroticola* disease outbreak.

### 3.2. Microbiome diversity

The method of denoising and generating feature sequences represented by DADA2 is strongly encouraged by current analysis platforms (QIIME2 and USEARCH). Here, all samples (*n* = 48) of the high-quality debarkation data were subjected to rigorous priming, quality filtering, denoising, splicing, mosaicking, and dereplication based on the DADA2 method (Callahan et al., 2016) to obtain ASVs (amplicon sequence variants). 16S rRNA variable region V3–V4 was amplified by PCR and high-throughput sequencing to obtain sequencing volume statistics for individual biological samples in Supplementary Table S1. Sequencing volume statistics of ITS were shown in Supplementary Table S2. As a result, the effective sequence coverage reached 99%. These results show the high reliability of the instrument test data. In addition, the curve of Shannon index finally tends to the level of 48 samples, confirming that the sequencing depth was sufficient to cover the whole diversity in the samples, and the diversity of each sample was measured to some extent by comparing the number of ASVs in the samples. The above data perfectly support the conclusion (Supplementary Figure S1).

Alpha diversity of the microbial community was assessed by species richness, evenness, and sequencing depth. Richness is represented by the Chao1 index and the Observed species index, Shannon and Simpson diversity index, Pielou’s evenness index, and Good’s coverage index (Supplementary Table S3 and Figure S2). The method for calculating the alpha diversity index can be found at http://scikit-bio.org/docs/latest/generated/skbio.diversity.alpha.html#module-skbio.diversity.alpha. Overall, the boxplot showed significant differences in the diversity of the different biological samples, and the significance *p* < 0.05 of Dunn’s test revealed the diversity information of the differences between the samples.

### 3.3. Taxonomic composition and fluctuations of the core microbiome

Representation of the taxonomic panorama of the samples at different classification levels by a limited framework. To represent the taxonomic composition of the microbial communities, we used the form of a circular packing diagram, which is intuitive and descriptive, and supplemented it with a pie chart that gives an overview of the compositional proportions of the different taxonomic units in the different groups (Supplementary Figure S3). The different coloring of the ASV points was used to highlight the taxonomic composition of the microorganisms at that level, and the area of the origin in the circle was used to indicate the abundance of the taxa corresponding to the circle. The results showed that the microbial community composition in the test sample was rich in diversity. The fruiting bodies and artificial bed-log of the ZCE group showed disease-free characteristics, while the bodies of the FBE group showed severe disease.

The Proteobacteria, Firmicutes, Bacteroidetes, and Actinobacteria showed the highest relative abundance of all bacterial phyla (Supplementary Table S4). The relative abundance of bacterial phyla varied among samples. The Proteobacteria strain had the highest relative abundance in FBE (average 97.68%), followed by MAT (average 76.64%), PRI (average 57.97%), ZCE (average 57.48%), FUL (average 54.80%), OPE (average 45.96%), and lower in ZCL and FBL. However, the relative abundance of Firmicutes strain was greater in ZCL (average 98.65%) and FBL (average 92.16%) than in the other groups, medium in OPE, FUL, and PRI, and smaller in ZCE, FBE, and MAT. Additionally, the relative abundance of the phyla Bacteroidetes, Actinobacteria, Verrucomicrobia, and WPS-2 was greater in ZCE. In particular, the phyla of Proteobacteria was significantly increased in group FBE compared to ZCE and the other groups. The strain of Basidiomycota had the highest relative abundance (more than 96.20%) in ZCE, ZCL, FEL, FUL, PRI, and MAT, but only 54.97% in FBE. In addition, relatively higher abundance of Ascomycota (average 40.06%) and Ascomycota (average 2.46%) was observed in the FBE group (Supplementary Table S5). These results confirm that spraying with pathogen suspensions altered the microflora in the fruiting bodies and artificial bed-log.

**Figure 2:**
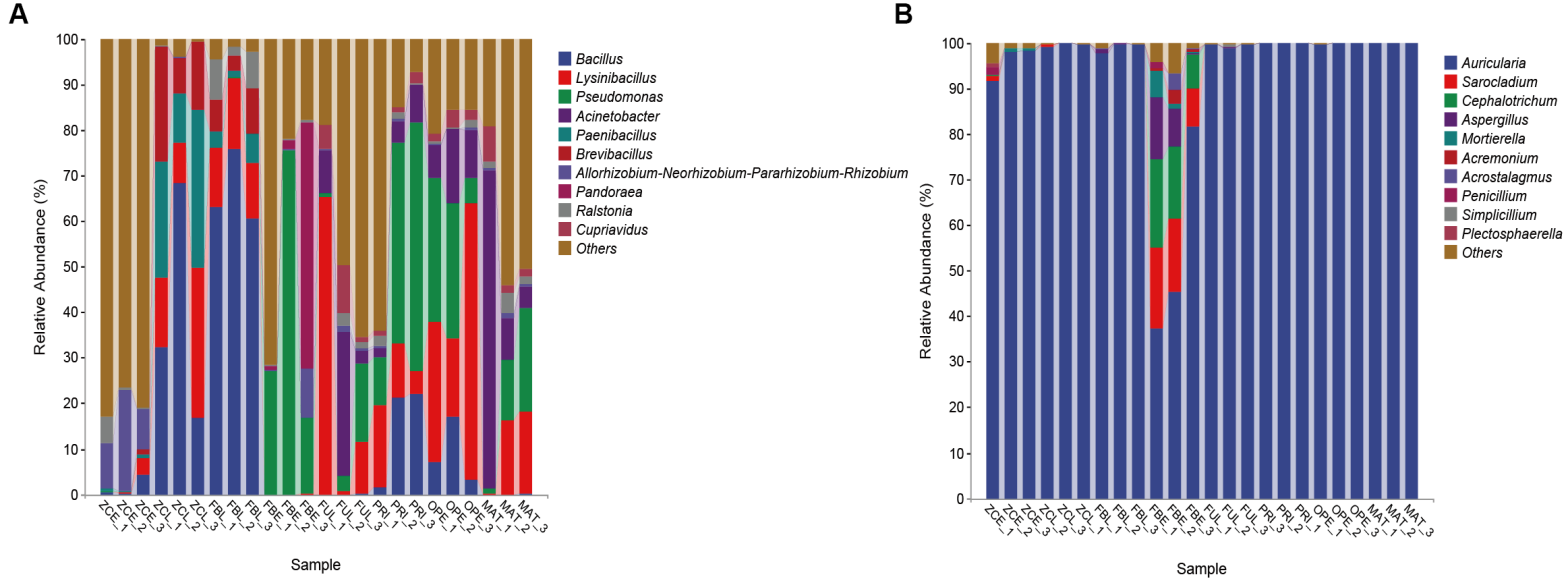
Taxonomic compositional variations over time at the genus level. **(A)** Bacterial communities. **(B)** Fungal communities. Sequences that could not be assigned to any known group were assigned to the “Others” category.

At the genus level, the 20 most abundant genera were displayed with relatively high abundance (Figure 2 and Table 1). The results show that the relative abundance of the genus *Bacillus* was higher in ZCL (average 39.19%) and FBL (average 66.52%), intermediate in PRI and OPE, and lower in ZCE, FBE, FUL, and MAT. The genus *Lysinibacillus* was most abundant in OPE (average 36.29%), followed by FUL, ZCL, and FBL, and least abundant in FBE and ZCE. Some species of *Pseudomonas* are considered to be widespread fungi causing diseases. In this study, the genus *Pseudomonas* was most abundant in the diseased body of FBE, intermediate in the medium of groups PRI, OPE, and MAT, and least abundant in ZCE, ZCL, and FBL. The genus *Acinetobacter* was more abundant in FUL, MAT, and OPE, but less abundant in ZCE, ZCL, FBL, and FBE. Overall, our results demonstrate the spatiotemporal dynamics of bacterial communities in fruiting bodies and artificial bed-log, as well as the succession of the dominant flora. Briefly, the genera *Lysinibacillus* and *Acinetobacter* were predominant in the FUL group. The genera *Bacillus* (except MAT), *Lysinibacillus*, *Pseudomonas*, and *Acinetobacter* were the dominant genera in the PRI, OPE, and MAT groups, respectively. The genera *Pseudomonas*, *Pandoraea* and *Pantoea* were dominant in FBE compared tovZCE. In the FBL group, the relative abundance of *Bacillus* and *Ralstonia* was higher and that of *Lysinibacillus*, *Paenibacillus*, and *Brevibacillus* was lower than in ZCL. It is noteworthy that *Pseudomonas*, *Pandoraea*, and *Pantoea* were relatively more abundant in the FBE group than in the other groups, which could be an important external reason for the rapid decay of the fruiting bodies.

**Table 1:** Relative abundance statistics of microflora at genus level (top 20, *n* = 3, %).

Microorganism	ID	ZCE	ZCL	FBL	FBE	FUL	PRI	OPE	MAT
Bacterial	*Bacillus*	1.70	39.19	66.52	0.01	0.04	14.92	9.18	0.04
	*Lysinibacillus*	1.21	18.95	13.58	0.01	25.82	11.65	36.29	11.44
	*Pseudomonas*	0.13	0.01	0.04	39.82	7.01	36.43	22.16	12.47
	*Acinetobacter*	0.00	0.00	0.00	0.00	14.78	4.94	11.41	27.92
	*Paenibacillus*	0.51	23.77	3.90	0.04	0.01	0.00	0.00	0.00
	*Brevibacillus*	0.39	16.00	6.76	0.00	0.00	0.00	0.00	0.00
	*Allorhizobium-Neorhizobium-Pararhizobium-Rhizobium*	13.74	0.02	0.00	3.74	0.60	0.39	0.29	0.72
	*Pandoraea*	0.00	0.00	0.00	18.95	0.00	0.00	0.00	0.00
	*Ralstonia*	2.21	0.20	6.29	0.35	1.44	1.34	0.86	2.41
	*Cupriavidus*	0.02	0.00	0.00	0.02	5.69	1.66	2.51	3.80
	*Pelomonas*	0.00	0.00	0.00	0.00	3.84	2.38	1.49	5.14
	*Pantoea*	0.00	0.00	0.01	11.08	0.00	0.00	0.00	0.01
	*Streptococcus*	0.00	0.00	0.00	0.00	4.75	3.60	0.08	0.25
	*Anoxybacillus*	0.00	0.00	0.00	0.00	1.27	1.07	3.66	0.84
	*Sphingomonas*	1.12	0.07	0.90	0.18	1.26	0.94	0.51	1.50
	*Burkholderia-Caballeronia-Paraburkholderia*	0.02	0.01	0.00	1.26	3.29	0.06	0.00	0.00
	*Flavobacterium*	4.17	0.05	0.00	0.31	0.00	0.01	0.01	0.02
	*Dyadobacter*	4.09	0.25	0.00	0.09	0.00	0.00	0.00	0.00
	*Vibrionimonas*	0.00	0.00	0.00	0.00	0.65	0.40	0.40	2.62
	*Williamsia*	3.56	0.00	0.00	0.00	0.00	0.00	0.00	0.00
	Others	67.14	1.48	2.00	24.13	29.55	20.20	11.15	30.83
Fungal	*Auricularia*	96.03	99.57	99.14	54.72	99.46	99.98	99.88	99.96
	*Sarocladium*	0.43	0.14	0.01	14.07	0.01	0.00	0.00	0.00
	*Cephalotrichum*	0.02	0.01	0.01	14.29	0.00	0.00	0.00	0.00
	*Aspergillus*	0.04	0.02	0.30	7.38	0.04	0.01	0.02	0.00
	*Mortierella*	0.34	0.00	0.00	2.46	0.00	0.00	0.00	0.00
	*Acremonium*	0.05	0.00	0.00	1.33	0.00	0.00	0.00	0.00
	*Acrostalagmus*	0.00	0.00	0.00	1.28	0.00	0.00	0.00	0.00
	*Penicillium*	0.59	0.05	0.00	0.54	0.00	0.00	0.00	0.00
	*Simplicillium*	0.00	0.00	0.00	0.00	0.23	0.00	0.00	0.00
	*Plectosphaerella*	0.23	0.00	0.00	0.00	0.00	0.00	0.00	0.00
	*Mycosphaerella*	0.17	0.00	0.00	0.03	0.00	0.00	0.00	0.00
	*Thermomyces*	0.00	0.00	0.00	0.14	0.00	0.00	0.03	0.00
	*Pseudocosmospora*	0.01	0.00	0.12	0.00	0.00	0.00	0.00	0.00
	*Alternaria*	0.11	0.00	0.00	0.00	0.00	0.00	0.00	0.01
	*Microascus*	0.00	0.00	0.00	0.11	0.00	0.00	0.00	0.00
	*Schizophyllum*	0.10	0.00	0.00	0.00	0.00	0.00	0.00	0.00
	*Thermoascus*	0.08	0.00	0.00	0.00	0.01	0.00	0.00	0.00
	*Hyphozyma*	0.00	0.00	0.00	0.07	0.00	0.00	0.00	0.00
	*Didymella*	0.06	0.00	0.00	0.00	0.00	0.00	0.00	0.00
	*Malassezia*	0.01	0.00	0.02	0.00	0.01	0.00	0.00	0.00
	Others	1.73	0.20	0.38	3.56	0.23	0.01	0.05	0.03

The FBE group had a markedly different composition of fungal communities, with greater relative abundance of *Sarocladium* (average 14.07%), *Cephalotrichum* (average 14.29%), *Aspergillus* (average 7.38%), *Mortierella* (average 2.46%), and a lower proportion of *Trichoderma* (less than 1%), the presence of which exacerbates body disease. However, the other groups all have extremely high abundances of *Auricularia*, which is consistent with the purpose of our study, and increases the satisfaction and credibility of the study data.

### 3.4. Differences and biomarker species

Differences in species composition between groups do not mean that there are differences in all species components, but often differences in the distribution of some components. In particular, some different components manifest themselves in different classification levels. The ASVs with statistically significant differences between sample groups were examined, and the enrichment trend of these differences at different classification levels was determined (Wippel et al., 2021). To further compare the differences in species composition between samples, the average abundance data of genera with the top 30 were used to draw heat maps (Supplementary Figure S4), showing the relative difference in abundance of core members between groups. Similarly, principal component analysis (PCA) was used to quantify the degree of difference in species composition between samples (Supplementary Figure S5). The result of the bacterial community analysis showed that the first two principal components explained 58.1% of the variation information. The ZCL and FBL groups were closer to each other, FBE was separated from the other groups, and the remaining groups were closer to each other. This indicates a similar bacterial community composition between the groups that are closer to each other. In the PCA analysis for fungi, cumulative 99.5% of the species information was interpreted by the first and second ordering axes, and the FBE group was clearly separated from the other groups, indicating that this group was clearly different from the other groups in microbial community, but the remaining groups had smaller distances, indicating that their microbiota composition was more similar to each other.

**Figure 3:**
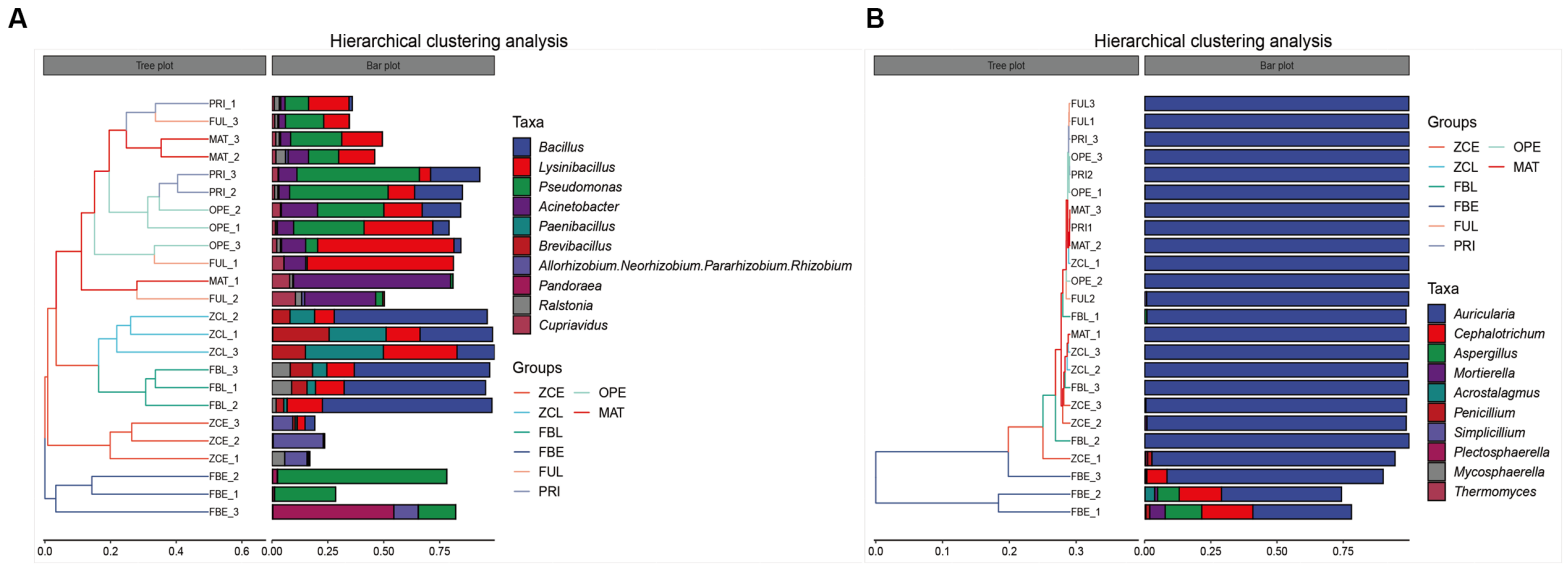
Hierarchical clustering analysis of **(A)** bacteria and **(B)** fungi communities. The panel on the left is a hierarchical clustering tree, in which samples are grouped by similarity. The shorter the branch length between the samples, the more similar they are. The right panel is a stacked bar chart of the top 10 genera in abundance.

At the genus level, the UPGMA algorithm (i.e., average clustering method) was used to perform cluster analysis using the Bray–Curtis distance matrix, and the similarity between samples was represented as a hierarchical tree (Figure 3). In Figure 3A, the hierarchical cluster tree on the left panel shows that FUL, PRI, OPE, and MAT are located in the same branch (i), indicating that their flora composition did not change significantly before spraying with the pathogen suspensions. FBL and ZCL are located on the same branch (ii). FBE is a single branch (iii) and ZCE is a single branch (iiii). The cluster tree shows that the bacterial communities in the fruiting bodies and artificial bed-log changed significantly after spraying with pathogens at MAT stage. The branch of (iii) represents high abundance of *Pseudomonas*, *Pandoraea*, and Pantoea. The branch of (ii) represents high abundance of *Bacillus*, *Lysinibacillus*, and *Paenibacillus*. The branch of (i) mainly shows the fluctuation of *Bacillus*, *Lysinibacillus*, *Pseudomonas*, and *Acinetobacter*. In Figure 3B, the hierarchical cluster tree on the left panel shows that the FBE cluster group is separated from the other groups, and the genus *Auricularia* is always the dominant genus, while *Sarocladium*, *Cephalotrichum*, and *Aspergillus* are particularly abundant. The results showed that spraying with the pathogen suspension had no significant effect on the microflora within the artificial bed-log, but significantly changed the composition of the fungal community in the disease body, decreased the relative abundance of the genus *Auricularia* and increased the relative abundance of *Sarocladium*, *Cephalotrichum*, and *Aspergillus*, etc. In general, the relative abundances of the genus *Auricularia* are always above 99% except for the ZCE group. The bar graph on the right shows the fluctuation data of the dominant genera in each sample, and is consistent with Figure 2.

### 3.5. Contribution and association network analysis of the dominant bacterial and fungal communities

**Figure 4:**
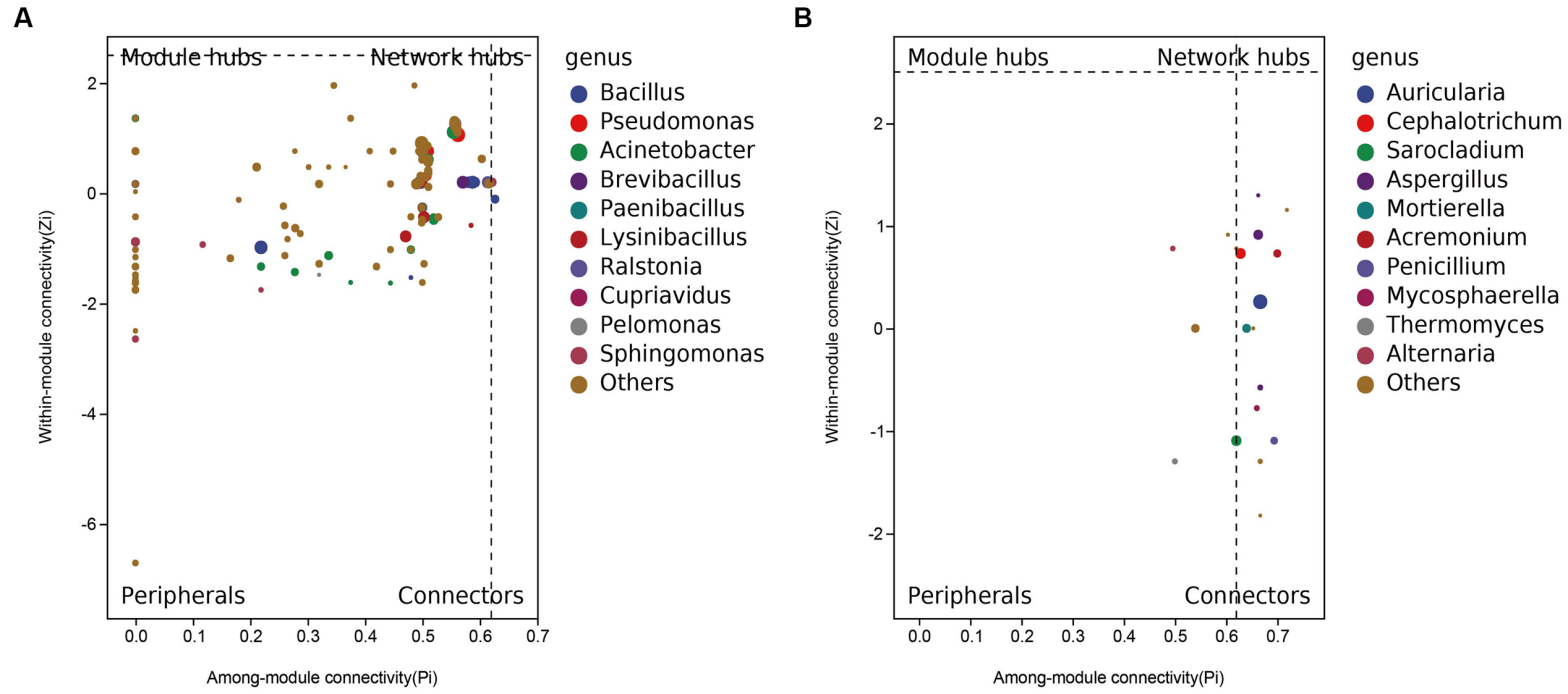
ZIPI plot of **(A)** bacteria and **(B)** fungi keystone species.

Inherent patterns of co-occurrence or co-exclusion of specific microbial communities, due to temporal and spatial changes and environmental processes, were searched for information on keystone species using association analysis. Species modules from all samples were combined into a network, and the 10 most abundant species modules were extracted for visual analysis to determine community relationships. Based on the SparCC algorithm, the correlation between community members was calculated using the microbial community composition data, and the correlation network was constructed (Supplementary Figures S6A,B). Furthermore, based on the abundance of nodes (ASV), the nodes with the 50 largest average abundances were extracted to create a subnetwork of dominant species, and visualized using the ggraph package (Supplementary Figures S6C,D). Using R, the within-module connectivity (Zi) and among-module connectivity (Pi) scores of each node in the current network are calculated based on the results of the modular intersection of the current co-occurrence network, and then the topological properties of each node in the associated network are determined according to the scores of Zi and Pi. The nodes (ASV) in the network are divided into four types, including peripherals (Zi < 2.5 and Pi < 0.62), connectors (Zi < 2.5 and Pi > 0.62), module nodes (Zi > 2.5 and Pi < 0.62), and network nodes (Zi > 2.5 and Pi > 0.62). The ZIPI plot perfectly shows the key species that play an important role in the succession of the predominant microbial community structure, namely the five major bacterial species (*Bacillus*, *Pseudomonas*, *Acinetobacter*, *Brevibacillus*, *Paenibacillus*) in abundance, and the fungal species *Auricularia*, *Cephalotrichum*, *Sarocladium*, *Aspergillus*, *Mortierella* (Figure 4). Overall, the changes in these key species are sufficient to cause changes throughout the microbiome.

In the four successive stages from FUL to MAT, the genera *Lysinibacillus*, *Pseudomonas*, *Acinetobacter*, and *Bacillus* (only on PRI and OPE) dominated in the culture medium. In contrast, the predominant bacterial community in the artificial culture medium changed significantly after the bodies were sprayed with the pathogen suspension at stage MAT. *Bacillus* (39.16 and 66.52%, respectively), *Lysinibacillus* (18.96 and 13.58%, respectively), *Paenibacillu* (23.77 and 3.90%, respectively), and *Brevibacillus* (16.00 and 6.76%, respectively) were the most abundant bacteria in the ZCL and FBL groups. The predominant fungal communities of healthy (ZCE) and diseased (FBE) fruiting bodies also showed significant differences. High abundance of *Allorhizobium-Neorhizobium-Pararhizobium-Rhizobium* was detected in the ZCE group (13.74%), but high abundance of *Pseudomonas* (39.82%), *Allorhizobium-Neorhizobium-Pararhizobium-Rhizobium* (3.74%), and *Pandoraea* (18.95%) was detected in the FBE group. In addition, the genus *Auricularia* (the relative abundance was over 99%) was the dominant fungal genus in the culture medium. The healthy fruiting bodies (ZCE) showed distinct fungal communities, and the relative abundance of the genus *Auricularia* was over 96.03%. However, the relative abundance of the genus *Auricularia* in the disease fruiting bodies (FBE) was only 54.72%, while *Sarocladium* (14.07%), *Cephalotrichum* (14.29%), *Aspergillus* (7.38%), *Aspergillus* (14.29%), *Aspergillus* (7.38%), and *Mortierella* (1.33%) were abundant. These additional microorganisms could be the main flora that further aggravated the disease of the fruit body. Overall, spraying with pathogens significantly altered the predominant microflora in the fruiting body and culture medium, leading to the appearance of disease on the fruiting body. The alteration of the core microbial community plays an important role in the succession of the dominant community. In this study, the relative abundance of the sprayed pathogenic fungi was less than 1%, suggesting that they may play a more important pathogenic role in the early stages of the bodies disease.

### 3.6. Differential analysis of metabolic pathways of core microbial communities

**Figure 5:**
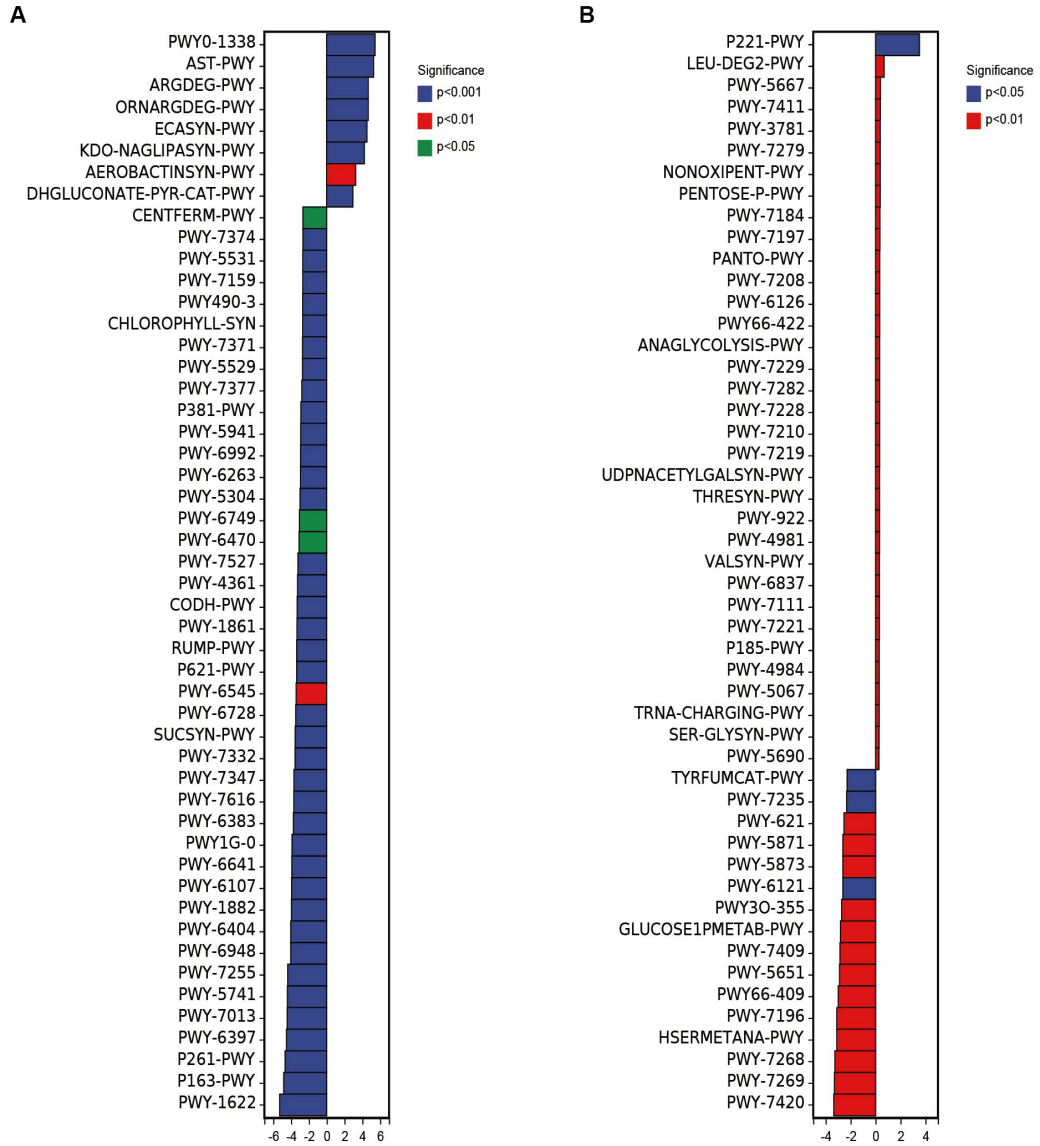
Differences in metabolic pathways of bacterial **(A)** and fungal **(B)** composition based on ZCE as control and FBE as up-regulation.

PICRUSt2 was used to predict 16S rRNA gene sequences in several functional databases (MetaCyc, KEGG, and COG), and ITS gene sequences were also used to predict metabolic pathways in MetaCyc databases. The metagenomeSeq method was used to predict the different metabolic pathways between groups (Figure 5). The results showed that 218 metabolic pathways were up-regulated in FBE compared with ZCE in predicting 16S rRNA gene metabolic pathways, including polymyxin resistance (PWY0-1338), L-arginine degradation II (AST pathway) (AST-PWY), superpathway of L-arginine, putrescine, and 4-aminobutanoate degradation (ARGDEG-PWY), superpathway of L-arginine and L-ornithine degradation (ORNARGDEG-PWY), glucose degradation (oxidative) (DHGLUCONATE-PYR-CAT-PWY), etc. Down-regulated 188 metabolic pathways, including formaldehyde assimilation I (serine pathway) (PWY-1622), L-lysine fermentation to acetate and butanoate (P163-PWY), coenzyme M biosynthesis I (P261-PWY), mycolyl-arabinogalactan-peptidoglycan complex biosynthesis (PWY-6397), etc. (Figure 5A). The glucose and glucose-1-phosphate degradation pathways in FBL were up-regulated compared with ZCL, and the superpathway of UDP-N-acetylglucosamine-derived O-antigen building block biosynthesis pathways was down-regulated. Predicting the ITS gene metabolic pathways, 51 metabolic pathways were up-regulated in FBE compared with ZCE (Figure 5B), including octane oxidation (P221-PWY), L-leucine degradation I (LEU-DEG2-PWY), CDP-diacylglycerol biosynthesis I (PWY-5667), superpathway of phosphatidate biosynthesis (yeast) (PWY-7411), superpathway of phosphatidate biosynthesis (yeast) (PWY-7411), etc. 20 metabolic pathways were down-regulated, including monoacylglycerol metabolism (PWY-7420), NAD/NADP-NADH/NADPH mitochondrial interconversion (PWY-7269), L-methionine biosynthesis III (HSERMETANA-PWY), superpathway of pyrimidine ribonucleosides salvage (PWY-7196), superpathway of purine nucleotide salvage (PWY66-409), L-tryptophan degradation to 2-amino-3-carboxymuconate semialdehyde (PWY-5651), etc. Phosphatidylglycerol biosynthesis I was significantly down-regulated in FBL compared with ZCL. Overall, spraying with pathogenic invasive microbes *T. pleuroticola* significantly altered the diversity of the microbial community in the fruiting body, resulting in a significant increase in metabolic pathways associated with reduced fruit quality. This study also confirmed that the pathogen is most likely introduced from the external environment to cause fruit body disease, supporting data for disease prevention and control.

## 4. Discussion

The pandemic of microbial diseases is causing a serious decline in the yield and quality of edible mushrooms. Microbial disease prevention and control has always been a focus of edible mushroom research and industrial development. Conventional diagnostic methods based on culturable microorganisms are fraught with difficulties. Metagenomic methods allow comprehensive analysis of the microbial composition and structure of specific samples and are considered suitable for identifying specific samples of microorganisms without laboratory culture. Artificial culture of *A. cornea* is an open system, in which the microbial community and environmental factors affecting the fruiting bodies and medium change simultaneously. These changes affect the composition and function of the microbial community, which is closely related to the growth of *A. cornea*, and then influence physiological function. Here, we provide an overview of the changes in the microbiome caused by brown rot pathogens. Metagenomic sequencing techniques provide a spatio-temporal overview of the bacterial and fungal communities in the fruiting body and MCM, the succession of the core flora that plays an important role, and the metabolic pathways in which they may be involved. Satisfactory data and reliable conclusions are critical for improving knowledge of brown rot disease.

Our research team first reported the brown rot disease of *A. cornea* caused by *T. pleuroticola* (Ye et al., 2023). Briefly, the pandemic of this disease causes large crop losses, posing a significant threat to the sustainability of crop production. Several studies have shown that *T. pleuroticola* was responsible for causing production-related diseases in crops, such as *Ganoderma lingzhi*, *Pleurotus ostreatus*, *Cyclocybe aegerita*, *Pleurotus eryngii*, and *Agaricus bisporus* (Komoń et al., 2007; Bellettini et al., 2018; Innocenti et al., 2019). The detrimental impact of *T. pleuroticola* manifests through its contamination of both the fruiting body and the MCM, resulting in browned mycelium and inhibited fruiting body growth (Largeteau and Savoie, 2010; O’Brien et al., 2017). Moreover, *T. pleuroticola* exhibits parasitic behavior towards other fungi, giving rise to toxic metabolites, decomposing enzymes, and volatile organic compounds. These harmful substances cause a range of effects including mycelial dissolution, browning, wilting, and eventual death, leading to a decrease or even complete cessation of commercial production (Choi et al., 2010; Mukherjee et al., 2022). Details of how the genus *Trichoderma* parasitizes other fungi have been published, including the use of a biomimetic system, scanning and transmission electron microscopy and fluorescence microscopy (Inbar and Chet, 1992). The key enzymes facilitating mycoparasitism, including chitinases, β-glucanases, and proteases, have been identified (Vazquez-Garciduenas et al., 1998; Carsolio et al., 1999). The present study showed that the fungal community in MCM did not change significantly throughout the experimental period, and the relative abundance of the genus *Auricularia* consistently exceeded 99%, although a spore suspension of 10^5^/mL (200 mL/m^2^) was sprayed at the MAT stage. Therefore, it can be demonstrated that the pathogenic invasive microbes *T. pleuroticola* are most likely introduced from external habitats and not from MCM, and are a non-endogenous pathogen. It is effective to prevent brown rot by improving the external environment of *A. cornea* cultures. Due to the parasitism of *T. pleuroticola* (Mukherjee et al., 2022), we observed a 40% decrease in the abundance of the genus *Auricularia* in FBE, along with the occurrence of *Sarocladium* (14.07%), *Cephalotrichum* (14.29%), and *Aspergillus* (7.38%). Surprisingly, the relative abundance of the genus *Trichoderma* detected in the fruiting body was less than 1%. Most microorganisms change their relationship with the host at different stages of their life cycle or in response to changing environmental conditions (Newton et al., 2010). Here, the reason for these results may be that *Trichoderma* plays an important role in the early stages of parasitic infestation and the relative abundance of other competing fungi rapidly increases with exposure to the lesion, while the relative abundance of *Trichoderma* rapidly decreases. This differs from the results of our experiment with Koch’s postulates conducted in the laboratory and greenhouse over a short period of time, as sampling in this study was delayed by 4 days, and the test was conducted in a large wood ear producing area with a higher disease load.

Bacterial communities exhibited greater variation than fungal communities. *Pseudomonas* and *Pandoraea* were high abundant in FBE compared to ZCE. However, *Allorhizobium-Neorhizobium-Pararhizobium-Rhizobium* were more abundant in ZCE than in FBE. Thus, spraying with the spore suspension of the pathogen significantly altered the bacterial community composition in the fruiting bodies and increased the relative abundance of *Pseudomonas* and *Pandoraea*. Some studies have shown that some *Pseudomonas* species are considered pathogenic to edible mushrooms. *Pseudomonas tolaasii* caused black rot of *Flammulina velutipes* in Korea (Han et al., 2012). *Pseudomonas yamanorum* caused blight disease of *Flammulina velutipes* (Wang et al., 2019). *Pseudomonas* spp. caused brown blotch of *Agaricus bisporus* (Abou-Zeid, 2012). *Pseudomonas* spp. strain P7014 and its supernatant in the mushroom culture media of *Pleurotus eryngii* resulted in faster growth of fungal mycelia, and a lower total number of cultivation days (5 ± 2 days) (Kim et al., 2008). Unfortunately, we have not found any useful data demonstrating that *Pseudomonas* spp. causes *Auricularia* disease. Therefore, we do not consider it a pathogen in the production system for *A. cornea* culture. *Pandoraea* has not been shown to cause mushroom diseases, and appears to be more associated with human health (Jørgensen et al., 2003; Lim et al., 2016). In MCM, bacterial communities show temporal and spatial variation, and higher abundance of *Bacillus*, *Paenibacillus*, and *Brevibacillus*, lower abundance of *Pseudomonas* and *Acinetobacter* in ZCL and FBL. *Bacillus* and *Paenibacillus* with higher abundance, *Brevibacillus* in ZCL and FBL, *Pseudomonas* with lower abundance and *Acinetobacter* in MCM showed spatial and temporal fluctuations. Similarly, *Bacillus*, *Paenibacillus*, and *Brevibacillus* have been shown to be the most important bacteria in MCM (Tan, 2019; Yang et al., 2022), supporting our view and lending credibility to the data. A multi-antibiotic resistant strain, *Paenibacillus* strain LK1 was isolated from the rhizosphere of *A. auricular* (Liu et al., 2018). Similarly, the genera *Bacillus*, *Paenibacillus*, *Brevibacillus* were the major bacteria in MCM (Tan, 2019; Yang et al., 2022), which supports our view and increases the credibility of the data. Moreover, our data show that *Lysinibacillus* was present in the whole MCM with a relative abundance ranging from 11.44 to 36.29%, which is consistent with the results of Tang (2019). Overall, our results improve the knowledge of brown rot caused by *T. pleuroticola*, and show a panoramic picture of the spatiotemporal dynamics of fungal and bacterial communities.

## 5. Conclusion

This study highlights the significant impact of the pathogenic invasive microbe *T. pleuroticola* on the microbiome composition and microecological processes in the *A. cornea* crop production system. Diseased fruiting bodies showed distinct microbial compositions, while core members in artificial bed-logs remained stable. *Pseudomonas* and *Pandoraea* bacterial genera, along with *Sarocladium*, *Cephalotrichum*, *Aspergillus*, and *Mortierella* fungal genera, were identified as biomarker species associated with fruit spoilage pathways after exposure to *T. pleuroticola*. Understanding these dynamics and interactions is crucial for disease control strategies and agricultural production improvement. Further research should explore the pathogen-host relationship and nutrient interaction process to enhance our knowledge and interventions for mitigating the impact of *T. pleuroticola*.

## Data availability statement

The datasets presented in this study can be found in online repositories. The names of the repository/repositories and accession number(s) can be found in the article/Supplementary material.

## Author contributions

LY: Conceptualization, Formal analysis, Software, Writing – original draft. BZ: Conceptualization, Formal analysis, Software, Methodology, Writing – original draft. LZ: Formal analysis, Methodology, Project administration, Software, Investigation, Writing – review & editing. XY: Formal analysis, Software, Data curation, Methodology, Project administration, Investigation, Writing – original draft. WT: Conceptualization, Funding acquisition, Resources, Supervision, Writing – review & editing. XZ: Funding acquisition, Methodology, Resources, Validation, Writing – review & editing. XL: Funding acquisition, Methodology, Resources, Validation, Writing – review & editing, Conceptualization, Project administration, Visualization.

## Footnotes

^1^ https://docs.qiime2.org/2019.4/tutorials/

^2^ https://github.com/QIIME2/q2-feature-classifier
