# Supplementary material for: Pathogenic invasive microbes Trichoderma pleuroticola transform bacterial and fungal community diversity in Auricularia cornea crop production system
Source: Front Microbiol. 2023 Nov 3;14:1263982. doi: 10.3389/fmicb.2023.1263982 (PMC10654786; doi:10.3389/fmicb.2023.1263982)
Supplement: Supplementary file 1 [file Data_Sheet_1.pdf]

Supplementary Material

Supplementary Figures

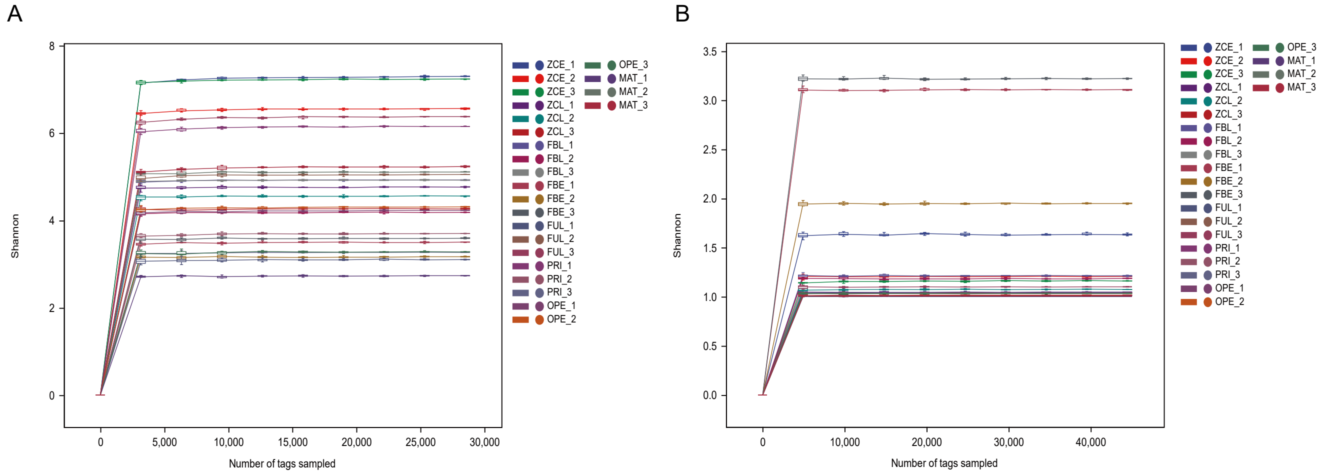

Fig. S1 Differences in rarefaction curves along the *A. cornea* crop production system for **(A)** bacterial and **(B)** fungal communities based on Shannon diversity index.

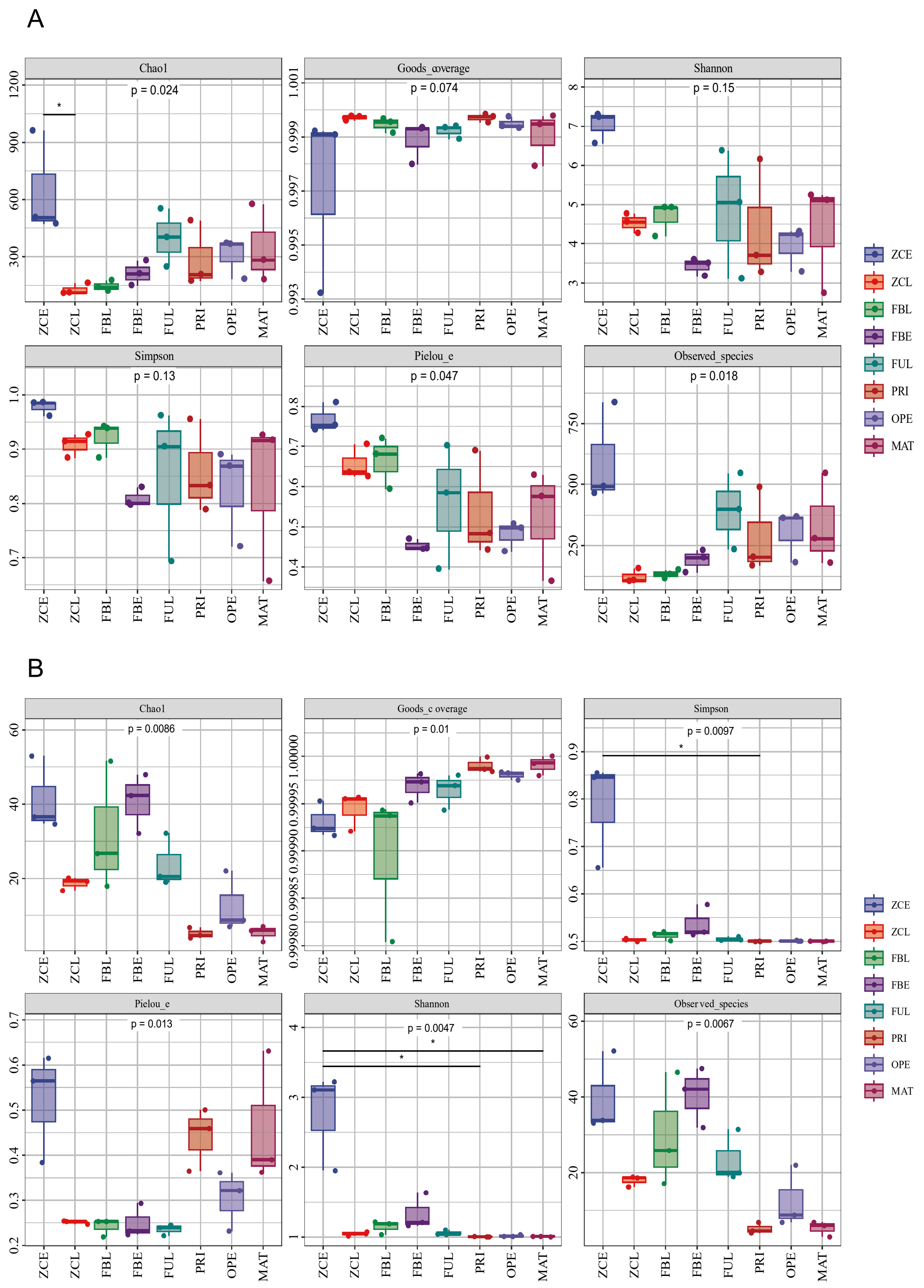

Fig. S2 Boxplots of alpha diversity indices. **(A)** bacterial diversity indices. **(B)** fungal diversity indices.

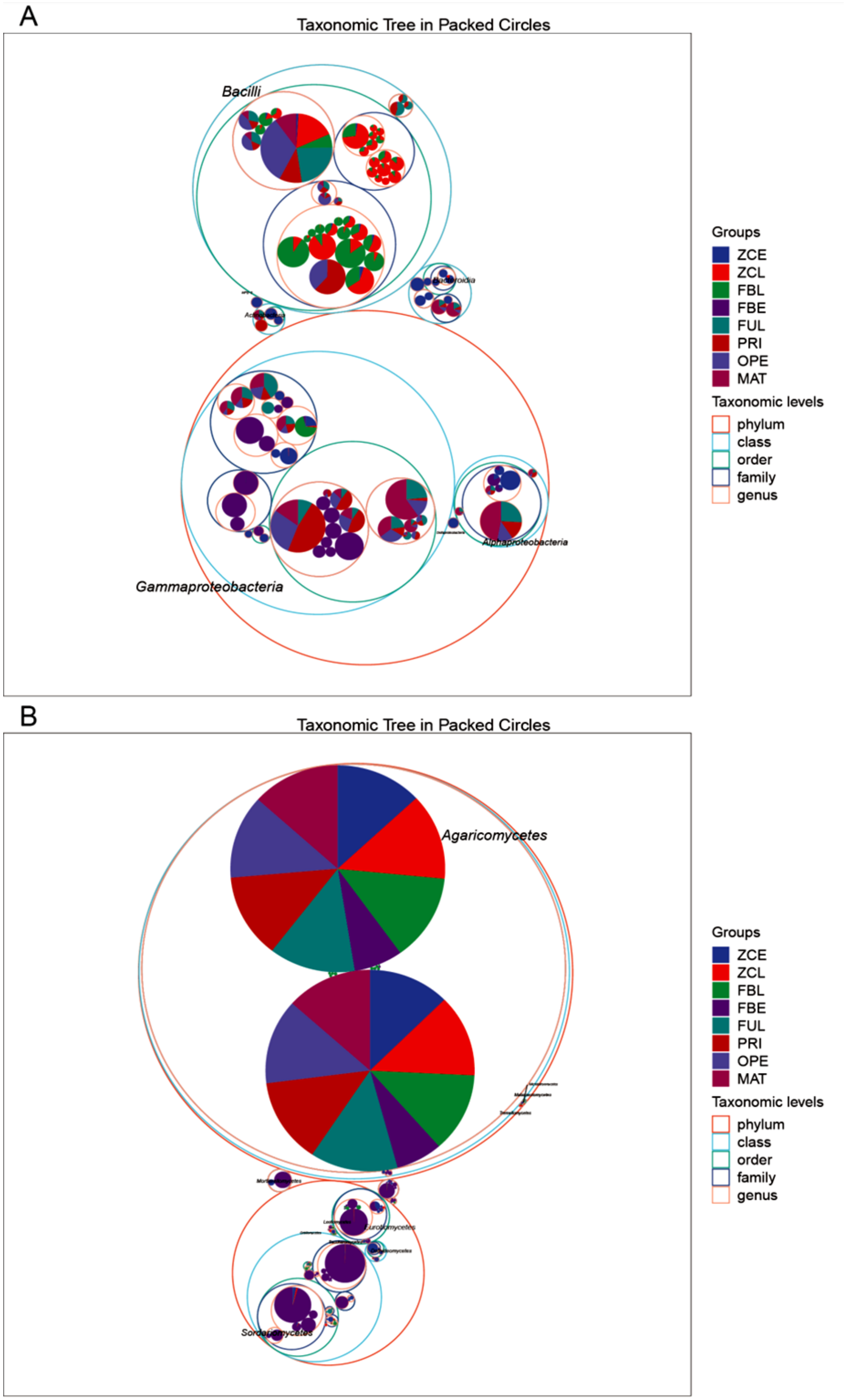

Fig. S3 Taxonomic tree in packed circles. **(A)** bacterial taxonomic tree. **(B)** fungal taxonomic tree. The largest circle represents the phylum level, and the decreasing circle represents the class, order, family, genus, and species in gradient order, using different colors to distinguish the different taxonomic levels. The innermost dot represents the top 100 ASVs in abundance, and its area is proportional to the abundance of that ASV.

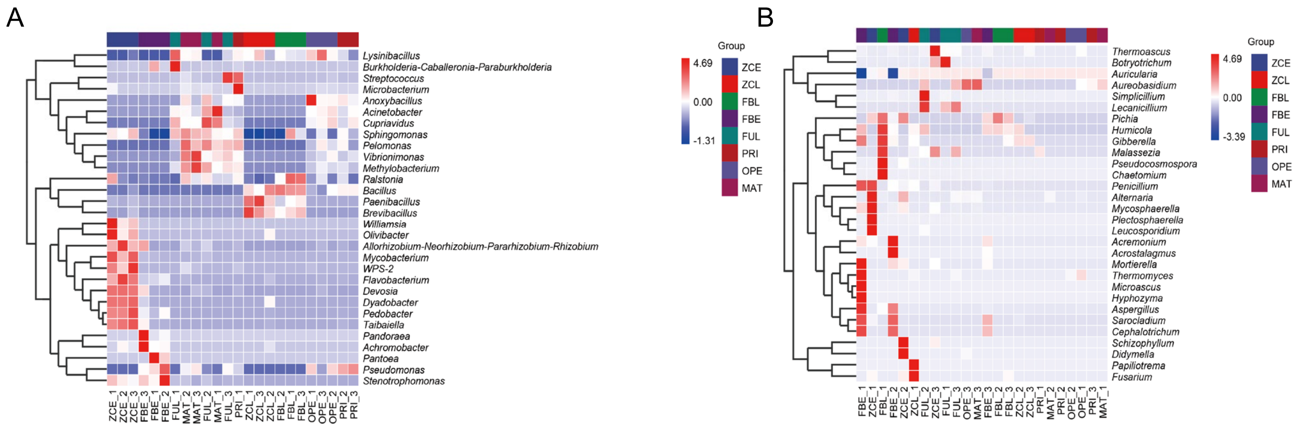

Fig. S4 Clustering heat map analysis of **(A)** bacterial and **(B)** fingal at genus level.

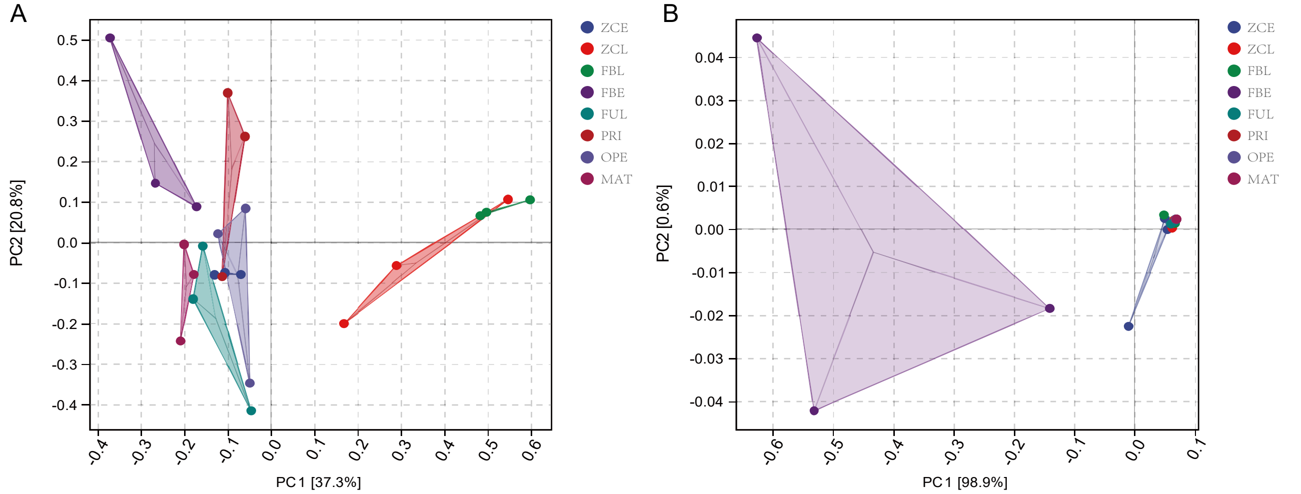

Fig. S5 Principal component analysis of **(A)** bacterial and **(B)** fungal community.

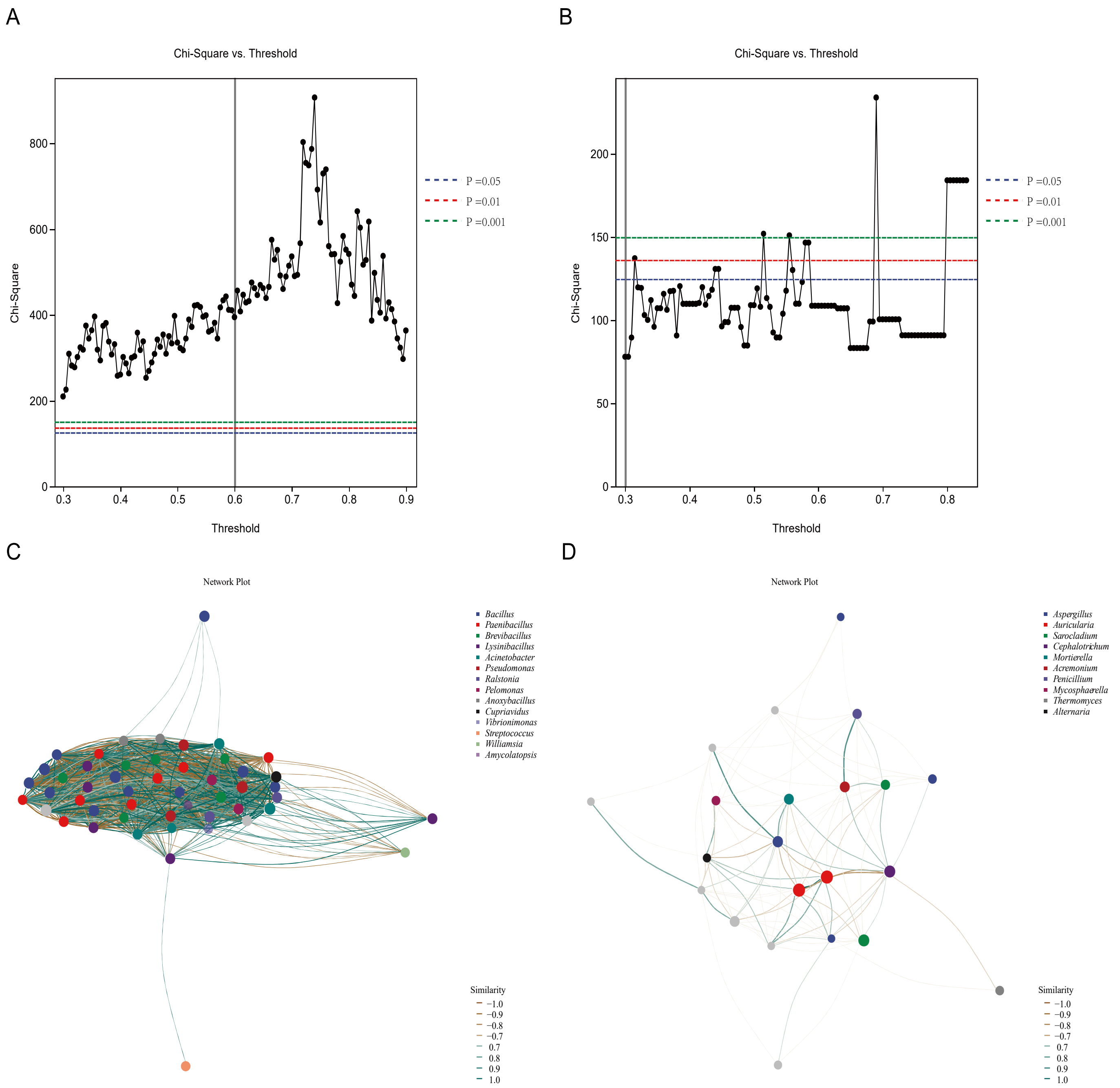

Fig. S6 Association network analysis. **(A, C)** based on bacterial community recognition data. The X and Y axes represent the correlation threshold (r) and the chi-square test (p), respectively. The green line represents the critical value of the chi-square test $p = 0.001$, the red line represents the critical value of the chi-square test $p = 0.01$, and the blue line represents the critical value of the Chi-square test $p = 0.05$. If p (r) is greater than the critical value, the r value at that time is selected as the correlation threshold for building the association network. **(B, D)** based on fungal community detection data. The node represents the ASV in the sample, the node size is proportional to its abundance ($\log_2$(CPM/n)), and the modules with the most nodes in the top 10 are labeled with different colors. The internode connection (edge) indicates that there is a correlation between the two connected nodes.

Supplementary Table

Table S1 Sequencing volume statistics of 16SrRNA (mean ± SD).

SampleID	Input	Filtered	Denoised	Merged	Non#chimeric	Non#singleton
ZCE	91767.00 ± 14553.84ab	73711.33 ± 11713.86a	72687.67 ± 10700.49a	69538.67 ± 6658.24a	63178.33 ± 5720.13a	62984.00 ± 5966.32b
ZCL	86426.00 ± 1623.05ab	69762.33 ± 2229.62a	69325.00 ± 2219.38a	68810.33 ± 2154.37a	63051.00 ± 6608.99a	63037.67 ± 6609.81b
FBL	99918.66 ± 27496.56ab	81430.00 ± 23253.85a	80879.00 ± 23107.11a	80146.33 ± 22870.99a	64029.67 ± 19739.87a	64001.67 ± 19739.46b
FBE	156794.00 ± 89044.97a	122526.33 ± 76263.24a	121999.67 ± 76163.74a	121442.00 ± 76037.11a	117076.67 ± 71267.15a	117051.33 ± 71255.63a
FUL	91438.67 ± 28386.94ab	83678.00 ± 22720.00a	82496.00 ± 23018.90a	80826.67 ± 23498.34a	77730.00 ± 24637.23a	38840.00 ± 12330.57b
PRI	74816.67 ± 7579.85b	70566.00 ± 7668.87a	69977.33 ± 7626.94a	69414.00 ± 7562.72a	67960.00 ± 6378.67a	33970.67 ± 3190.98b
OPE	79770.67 ± 5188.87b	75754.00 ± 4783.75a	75194.44 ± 5171.37a	74533.33 ± 5567.09a	73126.67 ± 6747.12a	36551.00 ± 3382.57b
MAT	99399.33 ± 35758.73ab	91242.67 ± 28841.72a	89267.33 ± 27444.55a	86237.33 ± 25038.94a	78368.00 ± 17497.62a	39146.33 ± 8711.68b

Input: the sequence volume of both forward and reverse primers that can be matched in the original data; **Filtered**: data volume after removing the low-quality sequence; **Denoised**: effective sequence volume; **Merged**: merged sequence volume; **Non#chimeric**: sequence volume after removal of chimeric, that is, high quality sequence quantity; **Non#singleton**: sequence volume after removing the singleton. ANOVA data were presented as mean ± standard deviation (±SD) of each sample group, n=3, $p \leq 0.05$ in LSD test. Means with the same lowercase letters are not significantly different. The same below.

Table S2 Sequencing volume statistics of ITS (mean ± SD).

SampleID	Input	Filtered	Denoised	Merged	Non#chimeric	Non#singleton
ZCE	122070.33 ±5713.42 b	103253.33 ± 14291.05	103134.00 ± 14294.08b	102527.67 ± 14290.36b	102211.00 ± 13744.77a	102211.00 ± 13744.77a
ZCL	127396.00 ± 3168.50ab	107808.00 ± 1445.95	107715.67 ± 1465.19ab	107192.00 ± 1601.40ab	107178.33 ± 1616.48a	107178.33 ± 1616.48a
FBL	135228.00 ± 5713.42ab	112314.33 ± 4594.06	112147.67 ± 4508.78ab	111010.00 ± 4541.40ab	110991.33 ± 4501.22a	110991.33 ± 4510.22a
FBE	138277.33 ± 12160.74a	118730.67 ± 10794.54	118561.67 ± 10755.02a	117831.33 ± 10591.30a	113456.67 ± 9409.54a	113456.00 ± 9408.97a
FUL	63149.00 ± 3762.09c	60124.67 ± 3393.77	60094.00 ± 3392.15c	60078.00 ± 3385.17c	60074.33 ± 3380.82b	60074.33 ± 3380.82b
PRI	62065.67 ± 2448.97c	59219.67 ± 2321.74	59210.67 ± 2324.27c	59203.00 ± 2318.84c	59203.00 ± 2318.84b	59203.00 ± 2318.84b
OPE	60581.00 ± 4758.31c	58105.33 ± 4593.58	57995.33 ± 4593.36c	57977.67 ± 4592.15c	57977.67 ± 4592.15b	57977.67 ± 4592.15b
MAT	53930.00 ± 5377.55c	51496.67 ± 5326.23	51483.00 ± 5315.67c	51479.00 ± 5311.73c	51479.00 ± 5311.73b	51497.00 ± 5311.73b

Table S3 Summary of number and diversity index of microbial communities at different sample stages (mean ± SD).

Microorganism	Index	ZCE	ZCL	FBL	FBE	FUL	PRI	OPE	MAT
Bacterial	Chao1	645.74 ± 272.70a	126.71 ± 30.32b	144.85 ± 28.17b	212.40 ± 65.01b	399.62 ± 152.49ab	288.59 ± 174.67b	306.86 ± 107.17b	346.86 ± 204.99b
	Shannon	7.03 ± 0.41a	4.53 ± 0.25b	4.68 ± 0.43b	3.42 ± 0.22b	4.84 ± 1.64b	4.37 ± 1.55b	3.94 ± 0.57b	4.36 ± 1.41b
	Simpson	0.98 ± 0.14a	0.91 ± 0.22a	0.92 ± 0.03a	0.81 ± 0.02a	0.85 ± 0.14a	0.86 ± 0.08a	0.83 ± 0.09a	0.83 ± 0.15a
	Observed species	597.47 ± 207.14a	122.87 ± 28.90c	132.53 ± 17.90bc	190.00 ± 45.82bc	392.40 ± 156.19ab	286.50 ± 176.04bc	303.33 ± 106.41bc	334.43 ± 189.69bc

Fungal	Chao1	40.77 ± 8.08a	18.66 ± 1.78bcd	32.12 ± 17.43ab	41.41 ± 10.07a	23.91 ± 7.26bc	5.13 ± 1.47d	12.64 ± 8.22cd	5.33 ± 2.08d
	Shannon	1.33 ± 0.26b	1.04 ± 0.03b	1.14 ± 0.09b	2.76 ± 0.70a	1.06 ± 0.04b	1.00 ± 0.00b	1.02 ± 0.01b	1.00 ± 0.00b
	Simpson	0.54 ± 0.03b	0.50 ± 0.00b	0.51 ± 0.01b	0.79 ± 0.11a	0.50 ± 0.00b	0.50 ± 0.00b	0.50 ± 0.00b	0.50 ± 0.00b
	Observed species	40.47 ± 7.91a	17.87 ± 1.45bcd	29.80 ± 15.10ab	39.67 ± 10.77a	23.43 ± 6.91bc	5.13 ± 1.47d	12.59 ± 8.22cd	5.30 ± 2.04d

Table S4 The relative abundance of bacterial phyla (top 10,%).

SampleID	Proteobacteria	Firmicutes	Bacteroidetes	Actinobacteria	Verrucomicrobia	WPS-2	Fusobacteria	Chloroflexi	Cyanobacteria	Deinococcus-Thermus	Others
ZCE	57.48	3.89	23.23	10.69	2.12	2.08	0.00	0.11	0.00	0.00	0.39
ZCL	0.69	98.65	0.57	0.05	0.00	0.00	0.00	0.02	0.00	0.00	0.02
FBL	7.71	92.16	0.12	0.00	0.00	0.00	0.00	0.00	0.00	0.00	0.01
FBE	97.68	0.13	1.84	0.21	0.09	0.00	0.01	0.00	0.00	0.00	0.04
FUL	54.80	36.52	2.07	3.62	0.11	0.00	0.68	0.45	0.26	0.39	1.11
PRI	57.97	34.39	1.49	4.50	0.02	0.02	0.62	0.09	0.18	0.06	0.66
OPE	45.96	50.85	1.48	0.57	0.02	0.01	0.01	0.14	0.09	0.36	0.51
MAT	76.64	13.78	5.81	2.06	0.02	0.06	0.03	0.23	0.38	0.08	0.92

Table S5 The relative abundance of the fungal phyla (top 4,%).

Sample ID	Basidiomycota	Ascomycota	Mortierellomycota	Mucoromycota	Others
ZCE	96.20	3.29	0.34	0.01	0.16
ZCL	99.63	0.35	0.00	0.00	0.02
FBL	99.20	0.74	0.00	0.00	0.05
FBE	54.97	40.06	2.46	0.00	2.50
FUL	99.50	0.44	0.00	0.00	0.06
PRI	99.98	0.02	0.00	0.00	0.00
OPE	99.89	0.10	0.00	0.00	0.02
NAT	99.96	0.04	0.00	0.00	0.00
